# Fibroblast activation protein-targeted near-infrared photoimmunotherapy depletes immunosuppressive cancer-associated fibroblasts and remodels local tumor immunity

**DOI:** 10.1038/s41416-024-02639-1

**Published:** 2024-03-30

**Authors:** Masaaki Akai, Kazuhiro Noma, Takuya Kato, Seitaro Nishimura, Hijiri Matsumoto, Kento Kawasaki, Tomoyoshi Kunitomo, Teruki Kobayashi, Noriyuki Nishiwaki, Hajime Kashima, Satoru Kikuchi, Toshiaki Ohara, Hiroshi Tazawa, Peter L. Choyke, Hisataka Kobayashi, Toshiyoshi Fujiwara

**Affiliations:** 1 Department of Gastroenterological Surgery, Okayama University Graduate School of Medicine, Dentistry and Pharmaceutical Sciences, Okayama, Japan; 2 Department of Pathology & Experimental Medicine, Okayama University Graduate School of Medicine, Dentistry and Pharmaceutical Sciences, Okayama, Japan; 3 Center for Gene and Cell Therapy, Okayama University Hospital, Okayama, Japan; 4 Molecular Imaging Branch, Center for Cancer Research, National Cancer Institute, National Institutes of Health, Bethesda, MD USA

**Keywords:** Cancer immunotherapy, Cancer microenvironment

## Abstract

**Background:**

Cancer-associated fibroblasts (CAFs) in the tumor microenvironment (TME) play a critical role in tumor immunosuppression. However, targeted depletion of CAFs is difficult due to their diverse cells of origin and the resulting lack of specific surface markers. Near-infrared photoimmunotherapy (NIR-PIT) is a novel cancer treatment that leads to rapid cell membrane damage.

**Methods:**

In this study, we used anti-mouse fibroblast activation protein (FAP) antibody to target FAP^+^ CAFs (FAP-targeted NIR-PIT) and investigated whether this therapy could suppress tumor progression and improve tumor immunity.

**Results:**

FAP-targeted NIR-PIT induced specific cell death in CAFs without damaging adjacent normal cells. Furthermore, FAP-targeted NIR-PIT treated mice showed significant tumor regression in the CAF-rich tumor model accompanied by an increase in CD8^+^ tumor infiltrating lymphocytes (TILs). Moreover, treated tumors showed increased levels of IFN-γ, TNF-α, and IL-2 in CD8^+^ TILs compared with non-treated tumors, suggesting enhanced antitumor immunity.

**Conclusions:**

Cancers with FAP-positive CAFs in their TME grow rapidly and FAP-targeted NIR-PIT not only suppresses their growth but improves tumor immunosuppression. Thus, FAP-targeted NIR-PIT is a potential therapeutic strategy for selectively targeting the TME of CAF^+^ tumors.

## Background

Cancer is a significant cause of morbidity and mortality throughout the world [1]. The main cancer therapies, surgery, chemotherapy, and radiotherapy, are widely used. However, recently a fourth therapy, immunotherapy, has been added to this list. For instance, immune checkpoint inhibitors (ICIs) have been used in multiple malignancies, resulting in the improvement of outcomes for many cancer patients [2–4]. Significantly, in 2020, the U.S. Food and Drug Administration (FDA) approved the use of pembrolizumab, an anti-programmed death receptor-1 (PD-1) antibody, as a primary therapeutic option for individuals with unresectable or metastatic microsatellite instability-high (MSI-H) or mismatch repair deficient (dMMR) colorectal cancer. As an illustration, the outcomes from the international phase II CheckMate 142 trial, focusing on colorectal cancer, demonstrated effectiveness specifically in cases with mismatch repair defects [5–7]. Consequently, immune checkpoint inhibitors (ICIs) such as anti-PD-1 and anti-cytotoxic T-lymphocyte-associated protein 4 (CTLA4) antibody therapies have become integral components of clinical practice [3, 8–10]. Despite the excitement surrounding ICIs, response rates remain low and immune-related adverse events (irAE) can cause serious harm. Systemic irAEs can cause critical and unexpected illnesses, but the mechanisms by which they occur and the target organs affected in an individual patient remain unclear. Thus, there are significant challenges to overcome before safe and effective immunotherapy is routine in the clinical setting. This will likely require a complete elucidation of the crosstalk between malignant tumors and their host environment.

The tumor microenvironment (TME) plays a major role in influencing tumor immunity. Among the diverse constituent cells of the TME, cancer-associated fibroblasts (CAFs) are commonly present and are thought to play essential roles in promoting tumor growth while suppressing immune surveillance [11–17]. We previously reported that the intra-tumoral accumulation of CAFs characterized by α smooth muscle actin (αSMA) and fibroblast activation protein (FAP) expression are associated with poor overall survival (OS) and disease-free survival (DFS) after radical esophagectomy and lymph node dissection [12, 14, 16]. CAFs also induced immunosuppressive tumor infiltrating lymphocytes (TILs), including CD8^+^ T cells and FoxP3^+^ T cells (regulatory T cells, Tregs) in both allograft tumor models and clinical esophageal cancer specimens [14]. Our previous investigations have revealed that CAFs within esophageal cancer tissue exert significant influences on various facets of tumor biology, including proliferation, angiogenesis, immunosuppression, migration, invasion, metastasis, and treatment resistance [11–15]. CAFs play a pivotal role in stimulating cancer cells and reshaping the TME through the secretion of signaling molecules [14, 17]. These include an array of inflammatory cytokines and growth factors, which collectively support tumorigenesis and the progression of the tumor [11, 15]. The exact effect of CAFs on tumor immunity remains unclear, however, cancer therapy targeting CAFs would appear to be a reasonable strategy to improve host antitumor immunity. However, currently there are no clinically approved CAF-targeted therapies. Novel therapies targeting CAFs are being developed that employ a variety of approaches; i) direct or indirect depletion of CAFs, ii) targeting the tumor-promoting and/or immunosuppressive functions of CAFs, or iii) reprogramming CAFs to a more quiescent state. [18] While these approaches are innovative, they are all systemic treatments with potential for on-target, off-tumor adverse events.

Near-infrared photoimmunotherapy (NIR-PIT) is a local targeted therapy that causes necrotic cell death by specifically binding antibody-photoabsorber conjugates (APC) to receptors on the target cell membrane and then exposing those cells to NIR light. NIR-PIT is highly selective and will not damage adjacent normal cells [19–26]. In a phase I/II clinical trial, epidermal growth factor receptor (EGFR)-targeted NIR-PIT was found to be safe and effective against unresectable or recurrent head and neck squamous cell carcinoma [27, 28]. Currently, antiEGFR NIR-PIT is in worldwide phase III clinical testing (SCC; LUZERA-301, NCT03769506) and has been conditionally approved in Japan in this clinical setting [16]. Originally, NIR-PIT was designed as a therapy that targeted cancer cells, but it was soon realized that it could be equally applied to other types of cells within the TME. CAFs therefore, are a reasonable target for NIR-PIT, and we previously established CAF-targeted NIR-PIT using FAP as a target which successfully and selectively depleted CAFs, resulting in tumor regression [13, 16, 29]. However, the impact of CAF-targeted NIR-PIT on tumor immunity has not been evaluated.

We now focus on the immune response after the localized killing of CAFs and hypothesize that FAP-targeted NIR-PIT reduces tumor immunosuppression as one mechanism of its anti-tumor activity. In this study, we established a murine tumor model that includes CAFs and treated it with FAP-targeted NIR-PIT. We analyzed the efficacy of this therapy and describe changes in tumor immunity after FAP-targeted NIR-PIT in a syngeneic mouse tumor model.

## Methods

### Mice and cell lines

Athymic female nude mice (BALB/c-*nu/nu*) and female C57BL/6 mice were purchased from Clea Inc (Tokyo, Japan). Animals were maintained in specific pathogen-free conditions in the animal laboratory at Okayama University. In animal experiments, each group was set up with five animals to be a sufficient sample size for statistical studies. In this study, we used murine cell lines of colon adenocarcinoma (MC38) and fibroblasts (MEF). MC38 was purchased from Kerafast (Boston, US). MEF was purchased from the American Type Culture Collection (ATCC, Manassas, VA, USA).

### Stimulation of fibroblasts

To prepare CM, MC38 cells were cultured with Dulbecco’s Modified Eagle Medium (DMEM) containing 2% fetal bovine serum (FBS) for 48 h. Supernatants were removed, filtered, and stored at −30 °C. MEF cells were cultured for 98 h with CM. We used stimulated fibroblasts (CAF^MC38^) with the culture media of MC38 cells in vitro. When comparing MEFs and CAFs, MEF cells were cultured in DMEM containing 2% FBS for 96 h before use.

### Western blotting

MEF fibroblasts, stimulated with MC38 cell conditioned media (CM) for 96 h, were examined for the expression of αSMA and FAP by Western blotting. Cells were homogenized and whole proteins extracted by centrifugation for 10 min at 4 °C. Samples containing 40 μg of protein were separated by electrophoresis on a polyacrylamide gel. Proteins were transferred to membranes and probed overnight at 4 °C with primary antibody. We used anti-αSMA antibody (D4K9N, 1:1000, Cell Signaling Technology, Danvers, USA), anti-FAP antibody (ab53066, 1:1000, Abcam, Cambridge, UK) and β-actin (A5441, 1:1000, Sigma-Aldrich, St. Louis, USA). Membranes were then washed in buffer and incubated with secondary antibodies for 1 h at room temperature (RT). After washing, membranes were visualized using LAS-4000 mini (FUJIFILM, Tokyo, Japan).

### Synthesis of IR700-conjugated anti-FAP antibody

The conjugation of dyes with monoclonal antibodies (mAbs) has been reported previously [29]. We purchased anti-mouse FAP recombinant antibody from Creative Biolabs (HPAB-0171-CN). In brief, anti-FAP antibody was incubated with IR700 (66.8 μg, 34.2 nmol, 5 mmol/L in DMSO) in 0.3 mol/L Na_2_HPO_4_ (pH 8.5) at 4 °C for 2 h. The mixture was purified on a Sephadex G50 column (PD-10; GE Healthcare, UK). The protein concentration was determined using a Bio-Rad protein assay kit (Bio-Rad, CA, USA) by measuring the absorption at 595 nm with spectroscopy. We abbreviate anti-murine FAP antibody conjugated IR700 as FAP-IR700.

### Immunofluorescence microscopy

MEF fibroblasts were seeded onto 96-well plates at 2.0 × 10^3^ cells/well and cultured in DMEM (Sigma-Aldrich) containing 15% FBS or CM from MC38 cells for 96 h. For co-cultures, MEF fibroblasts were seeded onto 96-well plates at 2.0 × 10^3^ cells/well and cultured in CM from MC38 cells for 72 h (CAF^MC38^), then MC38 cancer cells labeled with Cytotell^TM^ ultragreen (AAT Abioquet, Inc, California, USA) were added at 1.0 × 10^3^ cells/well and co-cultured with CAF^MC38^ for 24 h. These cells were treated with 20 μg/mL of APC for 1 h at 37 °C in the same manner described above. After treatment, cells were washed with PBS and propidium iodide (PI, 1:2000 dilution, Sigma-Aldrich) was added to identify dead cells. Cells were then irradiated with NIR light (20 J/cm^2^), and morphological changes were observed before and after treatment using a fluorescence microscope (IX83; Olympus, Tokyo, Japan).

### In vitro FAP-targeted NIR-PIT

MEF fibroblasts were seeded in in 96-well plates at 2 × 10^3^ cells/well and cultured for 24 h. To stimulate MEF, the medium was changed to CM from MC38 cells and cultured for another 96 h. The cells were treated with anti-FAP conjugated IR700 and incubated for 1 h at 37 °C. In all the wells, the media was replaced after the conjugate was added. Cells were irradiated with 690 nm laser light (BrixX695-2500UHP; Omicron-Laserage Laserprodukte GmbH, Rodgau, Germany), at a power density of 10 or 20 mW/cm^2^, as measured with an optical power meter (PM 100, Thorlabs, Inc., NJ, USA). Quantitative evaluation of cell viability was performed using the Cell Proliferation Kit II (XTT) (Roche Diagnostics, Rotkreuz, Switzerland), according to the manufacturer’s protocol.

### In vivo FAP-targeted NIR-PIT

MC38 (0.5 × 10^6^ cells) with and without MEF (0.5 × 10^6^ cells) were suspended in PBS (150 μL) and injected subcutaneously into the right flank of 6-week-old female BALB/c-*nu/nu* and C57BL/6 mice. To evaluate tumor growth, the diameter of each tumor was measured every 3 days. Tumor volume (mm^3^) was calculated using the formula: length × width^2^ × 0.5. The treatment mice were randomized and injected with 50 μg/body of anti-FAP conjugated IR700 intraperitoneally when tumors reached 50–100 mm^3^. On the next two days, the tumors were irradiated with NIR light at 50 J/cm^2^ (150 mW/cm^2^).

For T-cell depletion studies, anti-CD8α antibodies (BP0061; BioXcell, New Hampshire, USA) and anti-CD4 antibodies (BP0003; BioXcell) were injected intraperitoneally at 10 mg/kg per day before the first injection of APC, and every 3 days thereafter, for a total of four treatment rounds. The animals were euthanized via CO_2_, and serum and tumor tissue were collected for further analyses.

### Immunohistochemical analysis

Harvested subcutaneous tumors were formalin-fixed and paraffin-embedded. Sectioned tissues were then deparaffinized and soaked in 0.3% H_2_O_2_ in methanol at RT for 10 minutes to extinguish endogenous peroxidase activity. Antigen retrieval was performed by heating specimens in a sodium citrate buffer solution using a microwave. After cooling, sections were incubated in Peroxidase Blocking Reagent (Dako, Santa Clara, USA) for 10 minutes at RT. Sectioned tissues were incubated with primary antibody against CD8 (clone 4SM15, 1:100 dilution, eBioscience, San Diego, USA) or FoxP3 (clone FJK-16s, 1:100 dilution, eBioscience) or aSMA (A5228, 1:1,000 dilution, Sigma-Aldrich) or CD4 (clone 4SM95, 1:100 dilution, eBioscience) or TGF-β (ab215715, 1:100 dilution, Abcam, Cambridge, UK) for 60 minutes at RT. Following three 5-minute washes with PBS, sections were incubated with secondary antibody for 30 minutes at RT. After washing, the enzyme substrate 3,30-diaminobenzidine (Dako, Santa Clara, USA) was used for visualization, and sections were counterstained with Meyer’s hematoxylin. Evaluation of sections were performed using ImageJ software. The number of cells expressing CD8, FoxP3, CD4 were counted in four randomly selected high-magnification fields. The scores of αSMA and TGF-β were evaluated using an “area index,” calculated in low magnification fields.

### Flow-cytometric analysis

For cultured cells, cells were washed and incubated with monoclonal antibodies for 30 min at RT in PBS containing 2% FBS. We used fluorescence antibody for PD-L1 (APC anti-mouse PD-L1 antibody, #124312, 1:100 dilution, BioLegend, Inc, San Diego, USA), CD73 (PerCP/Cyanine5.5 anti-mouse CD73 antibody, #127214, 1:100 dilution, BioLegend), FAP (HPAB-0171-CN, Creative Biolabs, Inc, New York, USA). Cells were then washed and analyzed on a BD FACSAria III or FACSLyric (BD Biosciences, Inc, Franklin Lakes, USA).

For isolation of TILs, tumor tissues were dissected from the mice and TILs were harvested using BD Horizon Dri Tumor & Tissue Dissociation Reagent (TTDR, BD Biosciences, Inc), according to the manufacturer’s protocol. After removal of RBCs and after washing TILs and tumor cells, were incubated with monoclonal antibodies for 30 min at RT in PBS containing 2% FBS. After excluding dead cells by Zombie NIR^TM^ Fixable Viability Kit (#423106, 1:100 dilution, BioLegend), we used a fluorescent antibody for CD3 (APC anti-mouse CD3 antibody, #100236, 1:100 dilution, BioLegend), CD8a (FITC anti-mouse CD8a Antibody, #100706, 1:100 dilution, BioLegend), CD366 (BV421 anti-mouse CD366 Antibody, #119723, 1:100 dilution, BioLegend), and CD279 (PE anti-mouse CD279 Antibody, #135205, 1:100 dilution, BioLegend). Cells were then washed and analyzed on a BD FACSAria III.

For intracellular cytokine staining of TILs, tumors were harvested as described above, and lymphocytes were stimulated for 6 h in the presence of phorbol-12-myristate-13-acetate (PMA) 20 ng/mL, ionomycin 1 μg/mL, and Brefeldin A 0.5 μL/well at 37 °C. Next, cells were harvested and labeled with monoclonal antibodies. After excluding dead cells by Zombie Aqua™ Fixable Viability Kit (#423102, 1:100 dilution, BioLegend), we used a fluorescent antibody for CD8a (FITC anti-mouse CD8a Antibody, #100706, 1:100 dilution, BioLegend), TNF-α (BV421 anti-mouse TNF-α Antibody, #506327, 1:100 dilution, BioLegend), IL-2 (APC anti-mouse IL-2 Antibody, #503809, 1:100 dilution, BioLegend), and IFN-γ (APC/Cyanine7 anti-mouse IFN-γ Antibody, #505849, 1:100 dilution, BioLegend, Inc). Cells were then washed and analyzed on a BD FACSAria III.

### ELISA

Cell culture supernatants and mouse serum samples were assessed for the levels of TGF-β using appropriate ELISA kits (R & D Systems, Inc, Minneapolis, USA), according to the manufacturer’s protocol.

### Statistical analysis

All statistical analyses were performed using JMP software (SAS Institute, Cary, NC, USA). OS and DFS were calculated using the Kaplan–Meier method, with the log-rank test to compare subgroups. Hazard ratios (HRs) and 95% confidence intervals (CIs) for clinical variables were calculated using Cox proportional hazard regression in univariate and multivariate analyses. Spearman’s correlation was used to assess relationships between variables. For group comparisons, the Mann-Whitney test or Student’s *t* test was used. For multiple-group comparisons, analysis of variance with Tukey’s test was used. Statistical significance was set at *p* < 0.05.

### Study approval

This study was conducted in accordance with the Declaration of Helsinki’s ethical standards and the ethical guidelines for medical and health research involving human subjects. The use of clinical samples was approved and reviewed by the Ethics Review Board of Okayama University (No. 1801-023; Okayama, Japan). The experimental animal protocol was approved and reviewed by the Ethics Review Committee for Animal Experiments at Okayama University (OKU-2020166).

## Results

### Successful conjugation with anti-murine FAP antibody and IR700

To establish FAP-targeted NIR-PIT in a murine model, we first conjugated anti-FAP mAb to IR700 (FAP-IR700). Colloidal blue and fluorescence image demonstrated that the anti-murine FAP antibody was successfully conjugated with IR700 (Supplementary Fig. S1A). Flow-cytometric analysis also showed FAP-IR700 successfully bound cells. After blocking with excess anti-FAP antibody, fluorescence signal was attenuated. This suggested that the affinity of anti-murine FAP antibody was maintained after IR700 conjugation and that the binding was specific (Supplementary Fig. S1B).

### FAP-targeted NIR-PIT selectively killed FAP^+^ murine CAFs in vitro

**Fig. 1:**
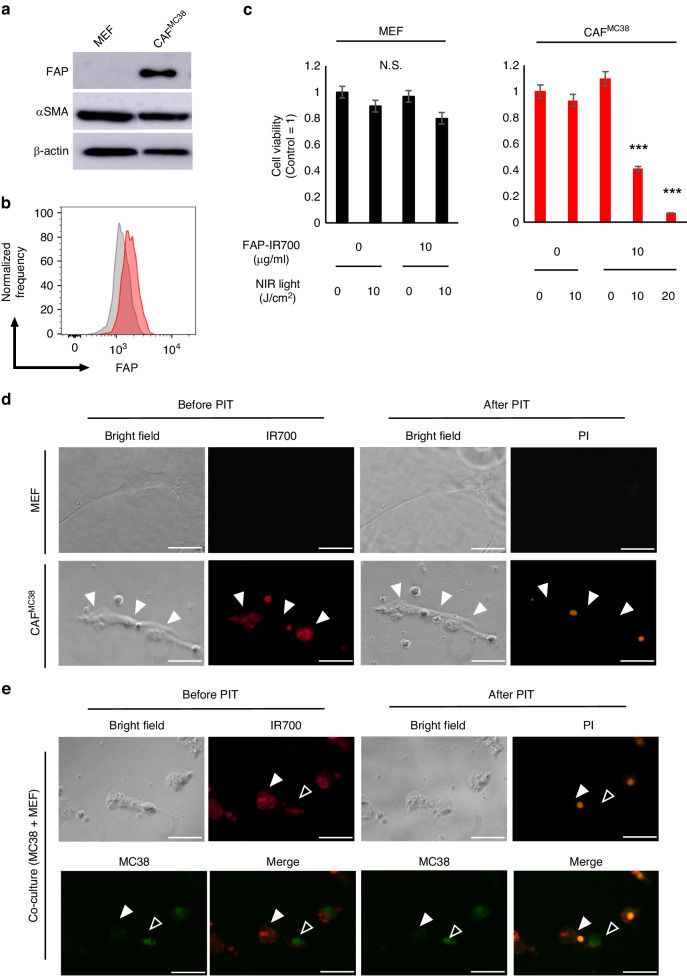
FAP-targeted NIR-PIT killed FAP^+^CAF selectively. **a** FAP or αSMA expression in MEFs by western blotting analysis. **b** Cell surface FAP expression on MEFs by flow cytometry. CAF^MC38^ was stimulated by conditioned medium from MC38 cells for 96 h and reacted with FAP-IR700. **c** The cell viability assay shows an inhibitory effect of FAP-targeted NIR-PIT on CAF^MC38^ in a dose- and light-dependent manner. Results for each experiment were compared with the control group. Significant inhibitory effects were revealed for the combinations of 10 J/cm^2^ and 20 J/cm^2^ NIR with 10 µg/mL FAP-IR700 (*n* = 5; mean ± SEM; ***P < .001, two-tailed Student’s *t* test.; N.S. not significant). **d** IHC microscopic images of fibroblasts. Activated fibroblasts expressed FAP (red) and were selectively killed by FAP-targeted NIR-PIT compared with normal fibroblasts (arrowheads). Scale bar = 50 μm. **e** IHC microscopic images of co-culture model (fibroblasts and cancer cells). MC38 cancer cells labeled with Cytotell^TM^ ultragreen (green, open arrowheads). Activated fibroblasts expressed FAP (red) and were selectively killed by FAP-targeted NIR-PIT (filled arrowheads). Scale bar = 50 μm. In **a** and **b**, representative examples from three experiments were presented, and in **c** and **e**, representative examples from five experiments were showcased.

To investigate the efficacy of FAP-targeted NIR-PIT, we stimulated fibroblasts (CAF^MC38^) with the culture media of MC38 cells in vitro. Western Blot analysis showed that CAF^MC38^ expressed FAP, while MEF did not (Fig. 1a). Flow-cytometric analysis also showed that CAF^MC38^ expressed FAP (Fig. 1b, Supplementary Fig. S2). These results suggested that normal fibroblasts were activated by MC38 cancer cells and upregulated their FAP expression. Next, the cytotoxic effect of FAP-targeted NIR-PIT was evaluated with the XTT assay. MEF and CAF^MC38^ cells were exposed to FAP-IR700 and then irradiated with NIR light. In CAF^MC38^, the cytotoxic effect was dependent on the dose of FAP-IR700 and the dose of NIR light. However, in normal fibroblasts (MEF) and cancer cells (MC38), there was no cytotoxic effect (Fig. 1c, Supplementary Fig. S3). Immunofluorescence microscopy showed that FAP-IR700 bound to CAF^MC38^ before NIR-PIT. Immediately after NIR irradiation, bleb formation was observed on the cell membrane of CAF^MC38^ which became swollen and necrotic beginning within 1 h of NIR irradiation; PI staining also supported cell death in CAF^MC38^ cells. However, these changes could not be observed in MEF or MC38 cells (Fig. 1d, e). Thus, FAP-targeted NIR-PIT could selectively kill CAFs without damaging adjacent normal cells.

### FAP^+^ CAFs promoted tumor growth in CAF-rich murine syngeneic models

**Fig. 2:**
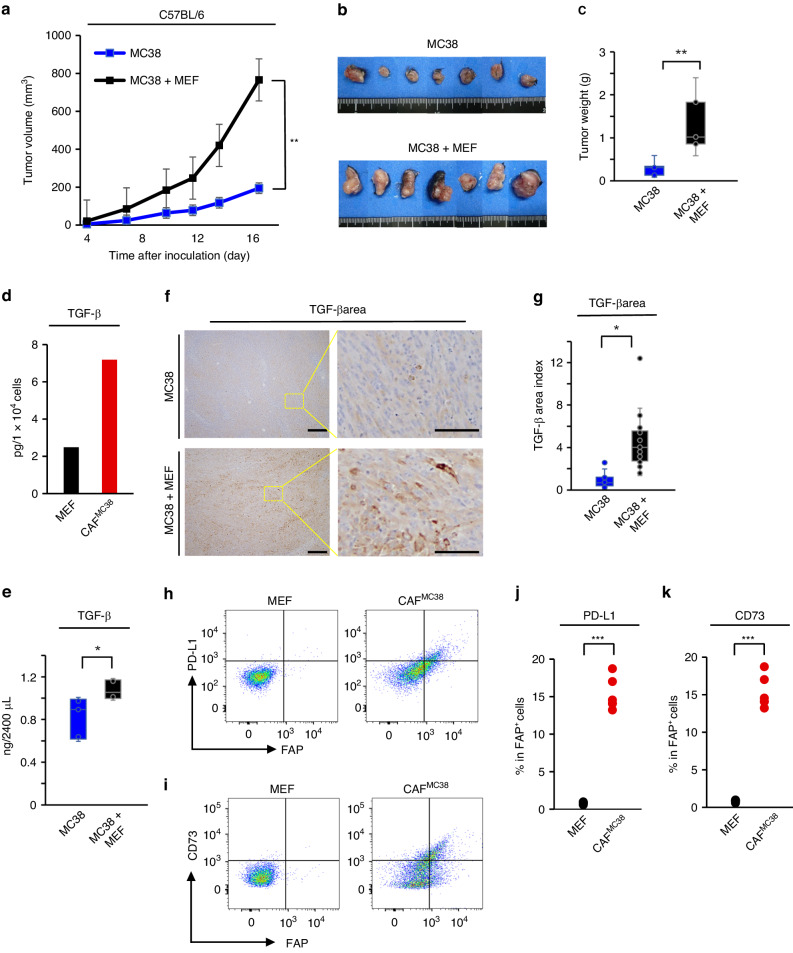
FAP^+^ CAFs promoted tumor growth and obtained immunosuppressive phenotype. **a** Tumor growth curve between MC38 + MEF tumors and MC38 tumor (*n* = 7, means ± SEM; **P < 0.01; Student’s *t* test on day 17). **b** Macroscopic image of harvested tumors. **c** Tumor weight between MC38 + MEF tumors and MC38 tumor (*n* = 7, means ± SEM; **P < 0.01; Student’s *t* test om day 17). **d** TGF-β secretion in supernatant from MEF or CAF^MC38^ via ELISA. **e** TGF-β secretion in tumor-bearing mice (MC38 or MC38 + MEF tumors) via ELISA. (*n* = 7, means ± SEM; *P < 0.05; Student’s *t* test). **f** Immunohistochemical images of TGF-β in MC38 or MC38 + MEF tumors. Right images were magnified. Scale bar = 200 μm (left), 50 μm (right). **g** Quantification of TGF-β positive area index by Image J software. (*n* = 7, means ± SEM; *P < 0.05; Student’s *t* test). Representative images of flow cytometry gated with FAP and PD-L1 (**h**) or CD73 (**i**). **j**, **k** Positive proportions of PD-L1^+^ cells (**j**) or CD73^+^ cells in FAP^+^ cells for MEF or CAF^MC38^ cells by flow cytometry (*n* = 5, means ± SEM; ***P < 0.001; Student’s *t* test). Representative examples from three experiments were presented in **d**–**k**.

Tumor progression was evaluated using co-inoculated allograft models of MC38 and MEF cells in C57BL/6 mice. The co-inoculated groups (MC38 + MEF) demonstrated significantly higher tumor growth rates compared with MC38 groups without MEF, which indicated CAF-poor tumors had slower growth rates (Fig. 2a). At 17 days after inoculation, MC38 + MEF groups also had significant higher tumor weights than MC38 tumors alone (Fig. 2b, c).

To evaluate the difference of humoral factors produced by fibroblasts, quantitative analysis of TGF-β by ELISA and IHC was performed. In both supernatant and serum, TGF-β was released in higher amounts by FAP^+^CAFs in comparison to normal fibroblasts both in vitro and in vivo (Fig. 2d, e). TGF-β was found in high amounts within the tumor bed in CAF-rich tumors (Fig. 2f, g).

### FAP^+^ CAFs are immunosuppressive

To understand CAF induced immunosuppression, we evaluated CAF^MC38^ using two immunosuppressive markers, PD-L1 and CD73. In CAF^MC38^, FAP, PD-L1 and CD73 expression increased compared with normal fibroblasts (Fig. 2h–k, Supplementary Fig. S4). These markers are associated with immunosuppressive environments.

### FAP-targeted NIR-PIT suppressed tumor growth in CAF-rich syngeneic models

**Fig. 3:**
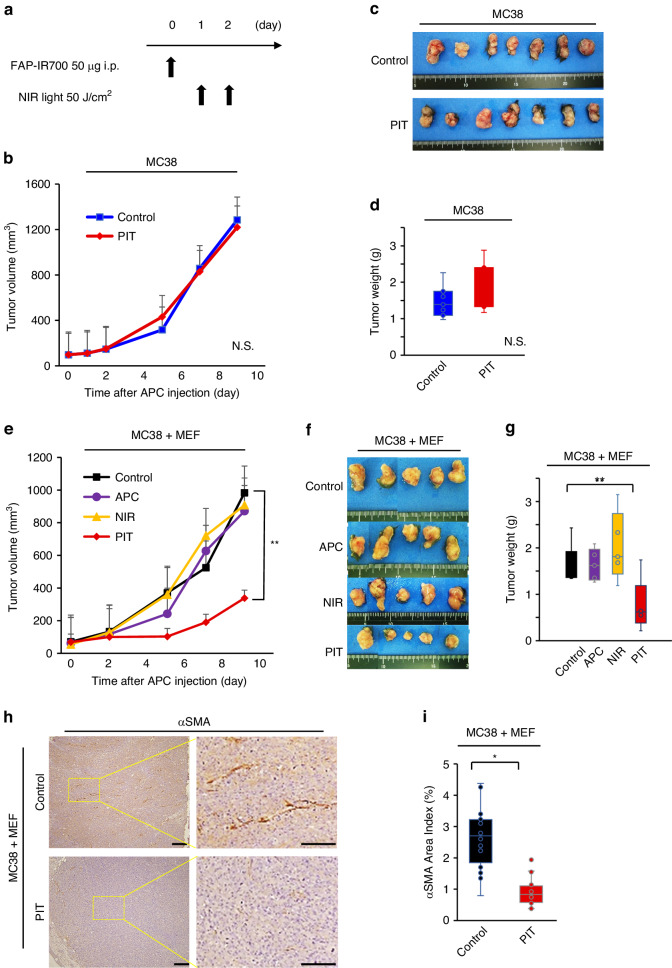
FAP-targeted NIR-PIT was effective in CAF-rich tumor model. **a** Therapy protocol in vivo is shown in Fig. 4a. **b** Tumor growth curve between the control and the FAP-targeted NIR-PIT groups on MC38 tumors (*n* = 7, mean ± SEM; N.S. not significant; Student’s *t* test on day 9). **c** Macroscopic image of harvested tumors for MC38 tumors. **d** Tumor weights for the control and the PIT groups (*n* = 7, means ± SEM; N.S. not significant; Student’s *t* test on day 9). **e** Tumor growth curve among the 4 groups; i) no treatment (Control), ii) treated with FAP-IR700 (APC), iii) irradiated with NIR light (NIR), and iv) treated with FAP-IR700 and irradiated with NIR light (PIT) on MC38 + MEF tumors (*n* = 5, means ± SEM; **P < 0.01; one-way ANOVA followed by Turkey’s on day 9). **f** Macroscopic image of harvested tumors for MC38 + MEF tumors. **g** Tumor weights among the 4 groups for MC38 + MEF tumors (*n* = 5, mean ± SEM; **P < 0.01; one-way ANOVA followed by Turkey’s on day 9). **h** Immunohistochemical images of αSMA in MC38 + MEF tumors. Right images were magnified. Scale bar = 200 μm (left), 100 μm (right). **i** Positive proportion of αSMA area index by Image J software. (*n* = 5, means ± SEM; *P < 0.05; Student’s *t* test).

As our previous studies demonstrated, CAFs contributed to tumor growth by inducing tumor immunosuppression [14, 29]. In this study, tumor growth was evaluated after FAP-targeted NIR-PIT in a syngeneic allograft model. The treatment regimen is shown in Fig. 3a. MC38 cells were inoculated into C57BL/6 mice in the right flank, mimicking CAF-poor tumors. The tumor-bearing mice were randomized as follows; i) no treatment (Control), ii) injected with FAP-IR700 followed by irradiation with NIR light (day 1 and 2, 50 J/cm^2^ each; PIT) (Fig. 3a). FAP-targeted NIR-PIT had minimal effect compared to the control group in CAF-poor tumor models (Fig. 3b–d).

NIR-PIT was next tested in a CAF-rich allograft model in which MC38 and MEF cells were simultaneously inoculated into C57BL/6 mice in the right flank. The tumor-bearing mice were randomized into 4 groups; i) no treatment (Control), ii) treated with FAP-IR700 (APC), iii) irradiated with NIR light (NIR), and iv) treated with FAP-IR700 and irradiated with NIR light (PIT). FAP-targeted NIR-PIT significantly suppressed tumor growth compared with the other groups (Fig. 3e). Tumor weights harvested 9 days after APC injection in the PIT group were also significantly decreased (Fig. 3f, g). IHC demonstrated that the αSMA positive staining area index was reduced significantly after FAP-targeted NIR-PIT (Fig. 3h,i). Body weights among all groups were not significantly different indicating an absence of systemic side effects (Supplementary Fig. S5A). In the APC alone and the NIR alone groups, no significant difference was detected, suggesting that neither APC nor NIR light alone affect this treatment (Fig. 3e, Supplementary Fig. S5B, C). These results suggest that FAP-targeted NIR-PIT causes tumor regression in a CAF-rich MC38 tumor model.

### Tumor growth suppression by FAP-targeted NIR-PIT was associated with heightened tumor immunity, exemplified by CD8^+^ tumor-infiltrating lymphocytes

**Fig. 4:**
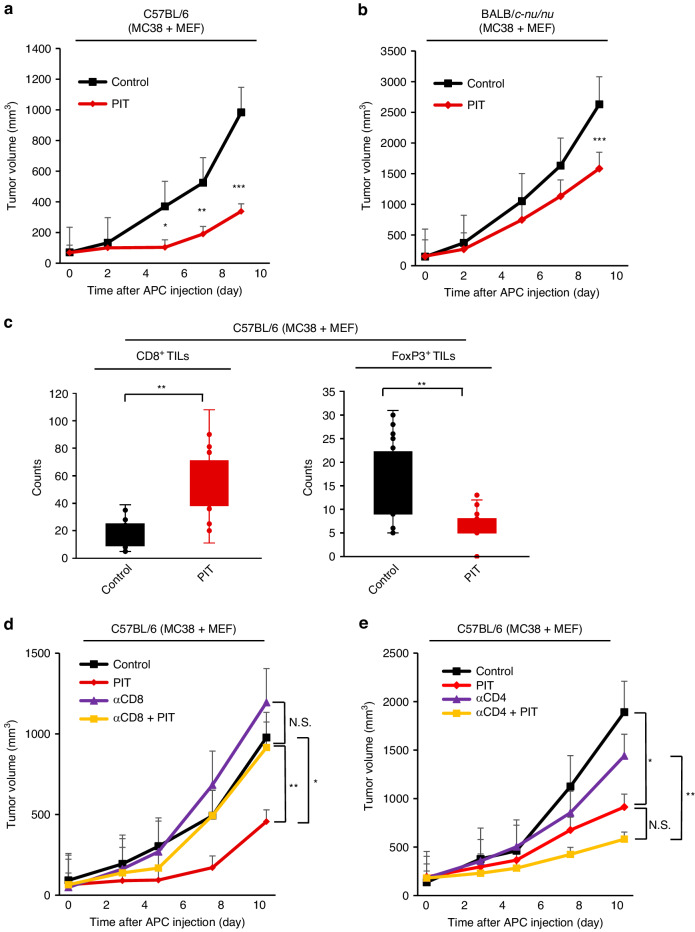
Tumor growth suppression by FAP-targeted NIR-PIT was associated with enhanced tumor immunity, especially with CD8^+^ tumor infiltrated lymphocytes. Comparison of tumor growth curves between the control and the FAP-targeted NIR-PIT (PIT) groups for MC38 + MEF tumor in C57BL/6 mice (**a**) or BALB/c nude mice (**b**) (*n* = 5, means ± SEM; *P < 0.05; **P < 0.01; ***P < 0.001; Student’s *t* test). **c** Quantitative analysis by IHC of CD8^+^ TILs and FoxP3^+^ TILs between the control and the PIT groups in C57BL/6 mice. IHC images were evaluated by Image J software. (*n* = 5, means ± SEM; **P < 0.01; Student’s *t* test on day 9). **d** Tumor growth curves among the control and the PIT groups with or without CD8^+^ T cells for MC38 + MEF tumor in C57BL/6 mice (*n* = 5, means ± SEM; *P < 0.05; **P < 0.01; Student’s *t* test). **e** Tumor growth curves among the control and the PIT groups with or without CD4^+^ T cells (*n* = 5, means ± SEM; *P < 0.05; **P < 0.01; Student’s *t* test).

To investigate the relationship between FAP-targeted NIR-PIT and host immunity, we evaluated the efficacy of NIR-PIT using immunocompetent mice (allograft model) and athymic mice (xenograft model), respectively. Tumor volume curves showed reduced tumor growth in the PIT groups in both mice tumor models. However, the anti-tumor effect of NIR-PIT in allograft models was observed earlier and was more robust compared to that in xenograft models (Fig. 4a, b). For instance, the reduction in tumor volume at day 9 after APC injection were 65.6% and 39.8% for allograft and xenograft models, respectively. This result demonstrated that the anti-tumor effect of FAP-targeted NIR-PIT strongly relied on a host tumor immunity.

After NIR-PIT, IHC of the remaining tissue showed that CD8^+^ T cell counts were increased significantly, and FoxP3^+^ regulatory T cells were decreased significantly after FAP-targeted NIR-PIT in allograft models (Fig. 4c). Another IHC result showed that TGF-β secretion decreased significantly after NIR-PIT (Supplementary Fig. 6). These results suggested that FAP-targeted NIR-PIT enhanced host antitumor immunity.

In addition, to investigate whether acquired immunity affected tumor growth suppression, CD4^+^ or CD8^+^ T cell depletion antibodies were administered prior to NIR-PIT in allograft models. The efficacy of NIR-PIT with anti-CD8 antibody pretreatment was greatly reduced, and no significant difference was observed between the control and anti-CD8 NIR-PIT groups (Fig. 4d). The efficacy of NIR-PIT pretreated with anti-CD4 antibody was unaffected and remained effective with or without anti-CD4 antibody (Fig. 4e). These results revealed that tumor growth suppression of FAP-targeted NIR-PIT is highly dependent on activated CD8^+^ TILs.

### FAP-targeted NIR-PIT gradually induced anti-tumor immune remodeling

**Fig. 5:**
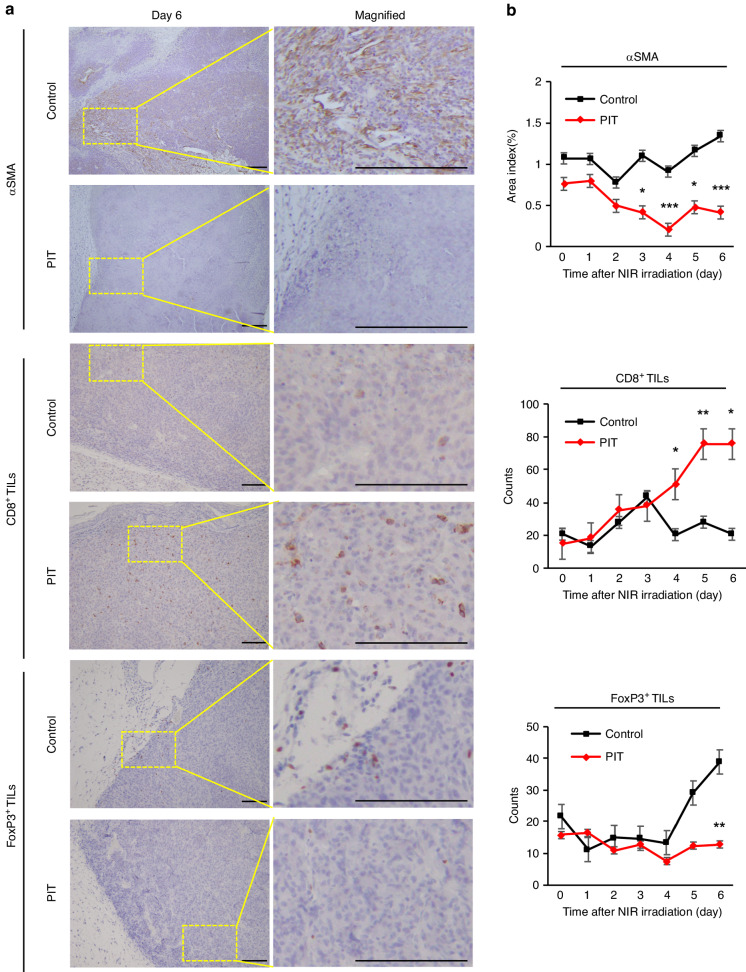
FAP-targeted NIR-PIT gradually induced tumor immunity remodeling. **a** Representative immunohistochemical images of αSMA, CD8^+^ TILs, and FoxP3^+^ TILs in the control and PIT groups on day 6. Rectangles were magnified images. Scale bar = 100 μm. **b** Temporal analyses for positive proportion of αSMA or quantification of CD8^+^ TILs and FoxP3^+^ TILs by immunohistochemical analysis (*n* = 5, means ± SEM; *P < 0.05; **P < 0.01; ***P < 0.001; Student’s *t* test).

To assess the temporal changes of FAP-targeted NIR-PIT on host tumor immunity, serial IHC of tumor tissue in allograft models was performed. The results showed that while αSMA positive areas began to decrease from day 3 and FoxP3^+^ cells began to decrease from day 5 after NIR-PIT, CD8^+^ T cells began to increase from day 4 after NIR-PIT (Fig. 5 and Supplementary Figs. S7–S9). These results suggested that FAP-targeted NIR-PIT began with a decrease in CAFs, followed by the induction of cytotoxic lymphocytes and coincident decrease in Tregs.

### FAP-targeted NIR-PIT activated host tumor immunity

**Fig. 6:**
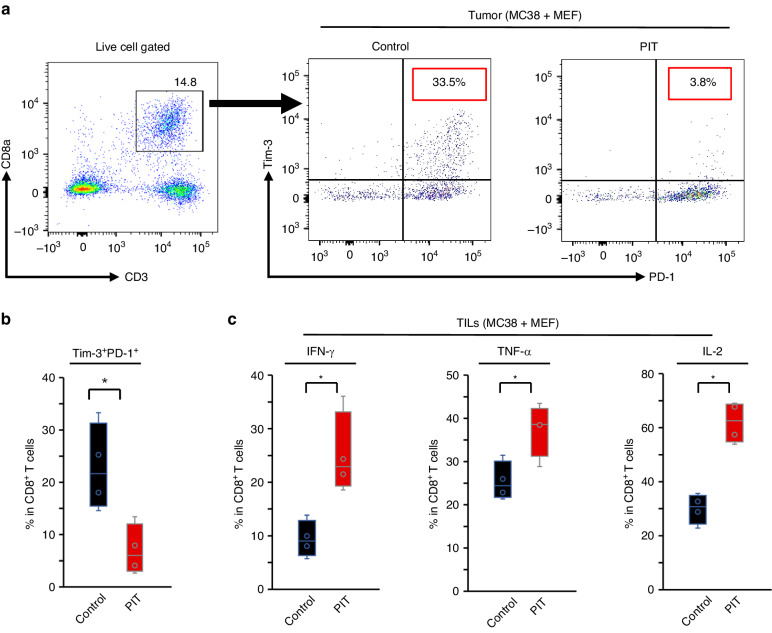
Tumor immune activation by FAP-targeted NIR-PIT depends on activation of tumor infiltrating CD8^+^ cells. **a** Gating strategy of TILs by flow cytometry. The percentage of Tim-3^+^ and PD-1^+^ in CD3^+^CD8^+^ within live lymphocytes were calculated. **b** The percentage of Tim-3^+^ and PD-1^+^ cells in CD8^+^ TILs for the control and the PIT groups (*n* = 4, means ± SEM; *P < 0.05; Student’s *t* test). **c** The percentage of IFN-γ^+^, TNF-α^+^ or IL-2^+^ cells in CD8^+^ TILs for the control and the PIT groups (*n* = 4, means ± SEM; *P < 0.05; Student’s *t* test). Representative examples from three experiments were presented in Fig. 6 for all experiments.

To evaluate the activity of TILs, first, T cell exhaustion markers with double positive PD-1 (CD279) and CD366 (Tim-3) in CD8^+^ T cells were evaluated. Terminally exhausted CD8^+^ T cells significantly decreased after FAP-targeted NIR-PIT (Fig. 6a, b, and Supplementary Fig. S10) whereas there was no significant difference in PD-1 positive CD8^+^ T cells (Supplementary Fig. S11). Next, to assess the activation of TILs, we evaluated cytotoxic markers INF-γ, TNF-α and IL-2. All of these markers in the PIT group were elevated compared to the control group (Fig. 6c). These results indicate that the tumor immune response was activated after FAP-targeted NIT-PIT.

## Discussion

In this study we demonstrated that murine FAP-targeted NIR-PIT could selectively deplete CAFs without damaging adjacent normal cells and suppress tumor growth in a CAF-rich tumor model. NIR-PIT resulted in an increase in the number and activity of CD8^+^ TILs while Tregs decreased in number and activity. Thus, FAP-targeted NIR-PIT induced TME immune remodeling resulting in activation of host tumor immunity and delay of tumor growth.

Because they can arise from several different cells of origin, markers that define CAFs are still open for debate. Conventionally, CAFs are defined as cells which express mesenchymal biomarkers, for example, vimentin, αSMA, platelet-derived growth factor receptor alpha (PDGFRα), and FAP [18]. Using FAP as a target for anti-CAF therapy we have shown that depletion of FAP^+^ CAFs has potent direct anti-tumor activity [13, 15, 16]. Additionally, we showed that FAP^+^ CAFs secrete TGF-β and lead to increased expression of PD-L1 and CD73, all markers associated with tumor immunosuppression (Fig. 3) [30–32]. Thus, FAP-expressing CAFs are immunosuppressive and aid the growth of tumor. Therefore, targeting FAP^+^ CAFs is a reasonable therapeutic strategy.

In this study, CAFs in allograft models were successfully targeted by NIR-PIT utilizing an anti-murine FAP antibody conjugated to IR700 dye. FAP-targeted NIR-PIT was more effective in immunocompetent mice than in athymic mice, suggesting that this therapy has both direct anti-cancer effects but also depends on activating the immune response by decreasing Tregs and increasing cytotoxic T cells in the TME. When CD8^+^ T cells were blocked with an anti-CD8 antibody, the immune response of NIR-PIT was significantly blunted. Collectively, these results suggest that selectively killing CAFs stimulated an immune response in the form of effector CD8^+^ T cells that infiltrated the TME and led to a reduced rate of tumor growth,

Sibrotuzumab, which is the first anti-FAP humanized monoclonal antibody, was developed as a CAF-targeted therapy to suppress tumor progression, however, this therapy has not yet shown clinical efficacy. This is likely because this agent has only been tested in recurrent tumors that have failed primary therapy and CAF-targeted therapy alone is unlikely to demonstrate efficacy with such aggressive tumors. Additionally, Tran et al. have reported that systemic depletion of FAP^+^ cells induced severe cachexy and bone toxicity [33]. Some of these limitations might be overcome with tumor selective CAF-directed depletive therapy. NIR-PIT can selectively kill targeted cells without damaging adjacent normal cells, therefore is a viable alternative to systemic CAF targeting. Moreover, local depletion of CAFs could lead to localized reprogramming of anti-tumor immunity and could activate an immune response without the systemic adverse effects of Sibrotuzumab. Since FAP is a reliable marker of CAFs it was chosen as the target for NIR-PIT.

Immune status within the tumor is important for predicting the response of immunotherapies [34, 35]. In the presence of FAP^+^ CAF cells, FAP-targeted NIR-PIT not only increased the number of CD8^+^ TILs but also enhanced their cytotoxicity. Furthermore, after FAP-targeted NIR-PIT, terminally exhausted PD-1^+^Tim-3^+^CD8^+^ TILs were decreased significantly, while PD-1^+^CD8^+^ TILs, which are reversibly exhausted CD8^+^ T cells, did not change in number. This raises the possibility that combining FAP-targeted NIR-PIT with an immune checkpoint inhibitor, like anti-PD-1 antibody, may induce synergistic effects to provoke reactivation of PD-1^+^CD8^+^ T cells. Although tumor progression by FAP-targeted NIR-PIT was strongly suppressed after only a single treatment, more intense treatment is needed to achieve complete remission in these models. As previously described, NIR-PIT has an inherent advantage because it can simultaneously target two or more kinds of cells by co-injecting two or more different APCs, or can be combined with other can also add other therapeutic modalities, such as immunotherapy [26, 36]. In addition, it has been demonstrated that cancer cell-targeted NIR-PIT also induces a host immune response [37]. Thus, the combination of FAP-targeted and cancer cell-targeted NIR-PIT has the potential to enhance each other’s therapeutic effects.

This study has several limitations. First, we used only one CAF-enriched allograft model. This is because such models require both an implanted tumor and CAFs derived from that tumor. It would be desirable to have other CAF^+^ mouse-derived cancer models. Also, BALB/c-nu/nu mice were used to examine the affection of host tumor immunity by FAP-targeted NIR-PIT because of familiarity and versatility. However, the species should be matched if strict acquired immunity is evaluated. Second, although we examined a number of known markers of immune suppression and activation, it is possible that additional features of the TME were also activated but were not investigated. More research is needed to clarify the mechanisms of action of FAP-targeted NIR-PIT. Furthermore, an orthotopic tumor model could offer a more clinically relevant representation by faithfully replicating the patients’ TME [38, 39]. Nevertheless, these models pose technical challenges when assessing the therapeutic effects in our experiments. Thus, further investigation is required.

In conclusion, we demonstrate that FAP-targeted NIR-PIT improved anti-tumor immunity in a CAF-rich tumor model, by selective killing FAP^+^ CAFs and activating an immune response. FAP-targeted NIR-PIT has great potential to overcome tumor immunosuppression and could be a novel therapeutic adjunct to cancer therapy in CAF^+^ tumors.

### Supplementary information

Supplemental Information

## Data Availability

The data generated in this study are available within the article and its Supplementary Data files.
