# Supplementary material for: Fibroblast activation protein-targeted near-infrared photoimmunotherapy depletes immunosuppressive cancer-associated fibroblasts and remodels local tumor immunity
Source: Br J Cancer. 2024 Mar 30;130(10):1647–58. doi: 10.1038/s41416-024-02639-1 (PMC11091110; doi:10.1038/s41416-024-02639-1)
Supplement: Supplementary file 1 — Supplemental Information [file 41416_2024_2639_MOESM1_ESM.docx]

Supplementary Information for

**Fibroblast Activation Protein-targeted Near-Infrared Photoimmunotherapy Depletes Immunosuppressive Cancer-Associated Fibroblasts and Remodels Local Tumor Immunity**

**Authors and affiliations:**

Masaaki Akai^1^, Kazuhiro Noma^1^, Takuya Kato^1^, Seitaro Nishimura^1^, Hijiri Matsumoto^1^, Kento Kawasaki^1^, Tomoyoshi Kunitomo^1^, Teruki Kobayashi^1^, Noriyuki Nishiwaki^1^, Hajime Kashima^1^, Satoru Kikuchi^1^, Toshiaki Ohara^1,2^, Hiroshi Tazawa^1,3^, Peter L. Choyke^4^, Hisataka Kobayashi^4^ and Toshiyoshi Fujiwara^1^

^1^ Department of Gastroenterological Surgery, Okayama University Graduate School of Medicine, Dentistry and Pharmaceutical Sciences, Okayama, Japan.

^2^ Department of Pathology & Experimental Medicine, Okayama University Graduate School of Medicine, Dentistry and Pharmaceutical Sciences, Okayama, Japan.

^3^ Center for Gene and Cell Therapy, Okayama University Hospital, Okayama, Japan.

^4^Molecular Imaging Branch, Center for Cancer Research, National Cancer Institute, National Institutes of Health, Bethesda, Maryland.

**Address correspondence to** Kazuhiro Noma, Department of Gastroenterological Surgery, Okayama University Graduate School of Medicine, Dentistry, and Pharmaceutical Sciences, 2-5-1 Shikata-cho, Kita-ku, Okayama 700-8558, Japan Phone: +81-86-235-7255; Fax: +81-86-221-8775; E-mail: [knoma@md.okayama-u.ac.jp](mailto:knoma@md.okayama-u.ac.jp)

**List of Supplementary Information**

Supplementary Figure S1. Quality check of anti-mouse FAP antibody conjugated IR700

Supplementary Figure S2. Gating strategy and FAP expression by flow cytometry in vitro

Supplementary Figure S3. The cell viability assay on MC38 after FAP-targeted NIR-PIT

Supplementary Figure S4. Gating strategy and expression of CD73 and PD-L1 for CAFs and normal fibroblasts by flow cytometry.

Supplementary Figure S5. Side effect of FAP-targeted NIR-PIT

Supplementary Figure S6. The effect for tumor microenvironment on MC38+MEF tumor after FAP-targeted NIR-PIT

Supplementary Figure S7. Representative immunohistochemical images of αSMA in the Control and PIT groups with several time course (day 0 to 5) in MC38+MEF allograft models.

Supplementary Figure S8. Representative immunohistochemical images of CD8^+^TILs in the Control and PIT groups with several time course (day 0 to 5) in MC38+MEF allograft models.

Supplementary Figure S9. Representative immunohistochemical images of FoxP3^+^TILs in the Control and PIT groups with several time course (day 0 to 5) in MC38+MEF allograft models.

Supplementary Figure S10. Gating strategy and representative flow cytometric histograms.

Supplementary Figure S11. The effect for tumor infiltrating lymphocytes on MC38+MEF tumor after FAP-targeted NIR-PIT

**Supplementary Figure S1**


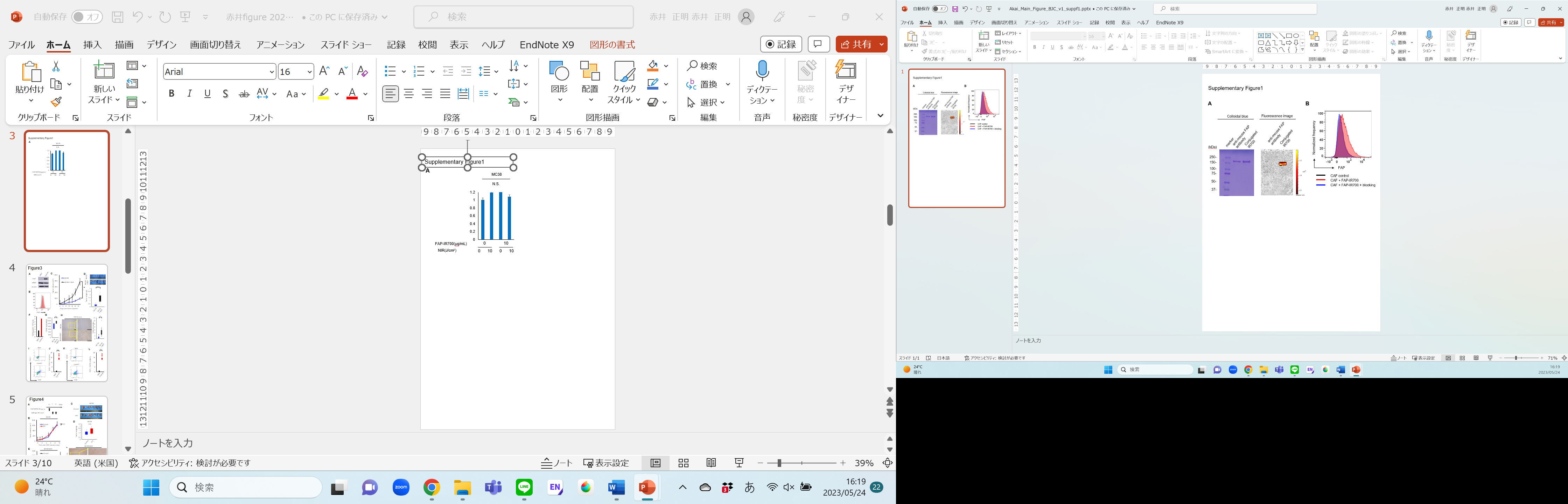


**Supplementary Figure S1. Quality check of anti-mouse FAP antibody conjugated IR700**

(A) Colloidal blue and fluorescence image after antibody electrophoresis. (B) Cell counts of FAP^+^ MEF cells with by flow cytometry.

**Supplementary Figure S2**


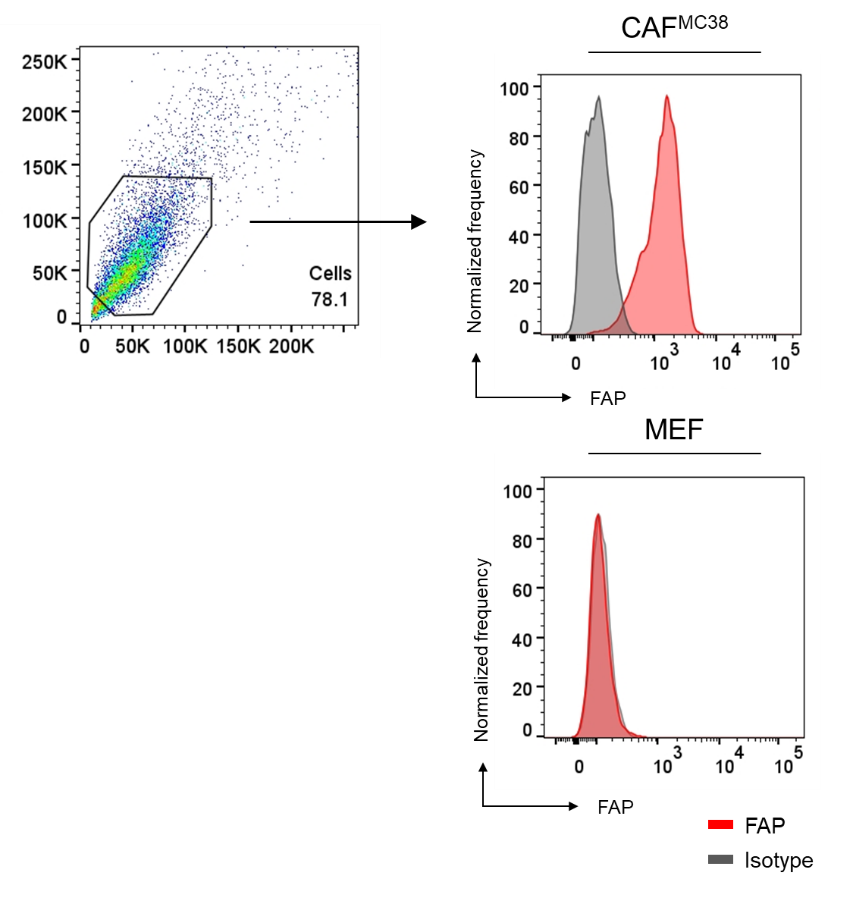


**Supplementary Figure S2.** **Gating strategy and FAP expression by flow cytometry in vitro.**

Gating strategy and representative flow cytometry plots of MEF and CAF^MC38^ cells with isotype controls in vitro.

**Supplementary Figure S3**


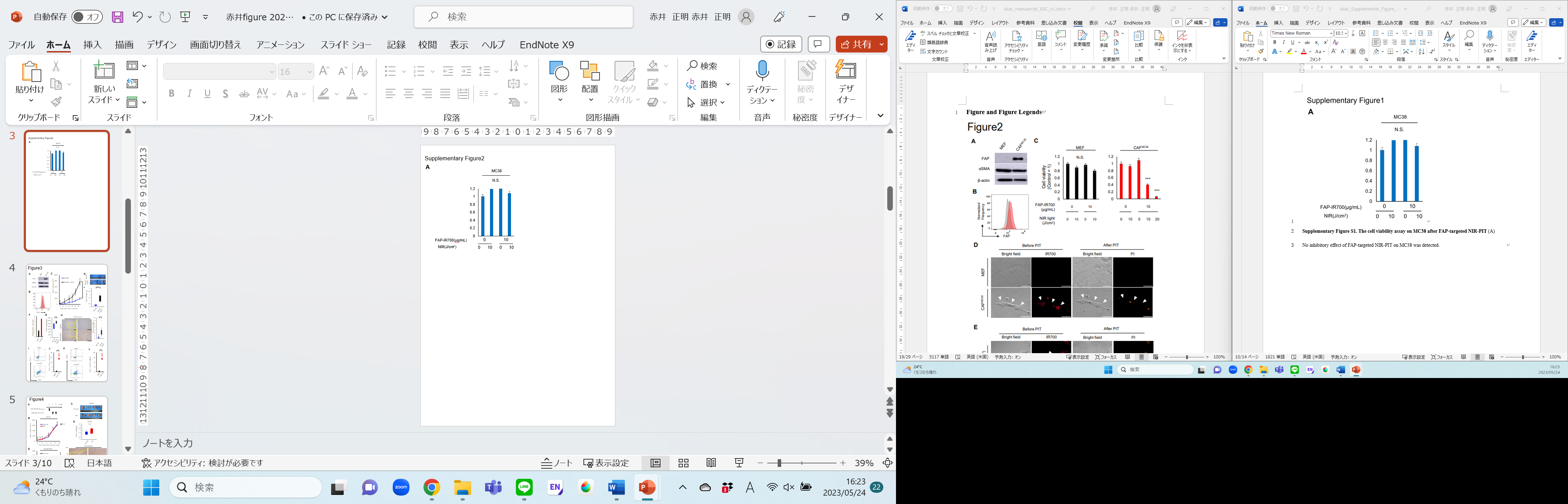


**Supplementary Figure S3. The cell viability assay on MC38 after FAP-targeted NIR-PIT.**

No inhibitory effect of FAP-targeted NIR-PIT on MC38 was detected.

**Supplementary Figure S4**


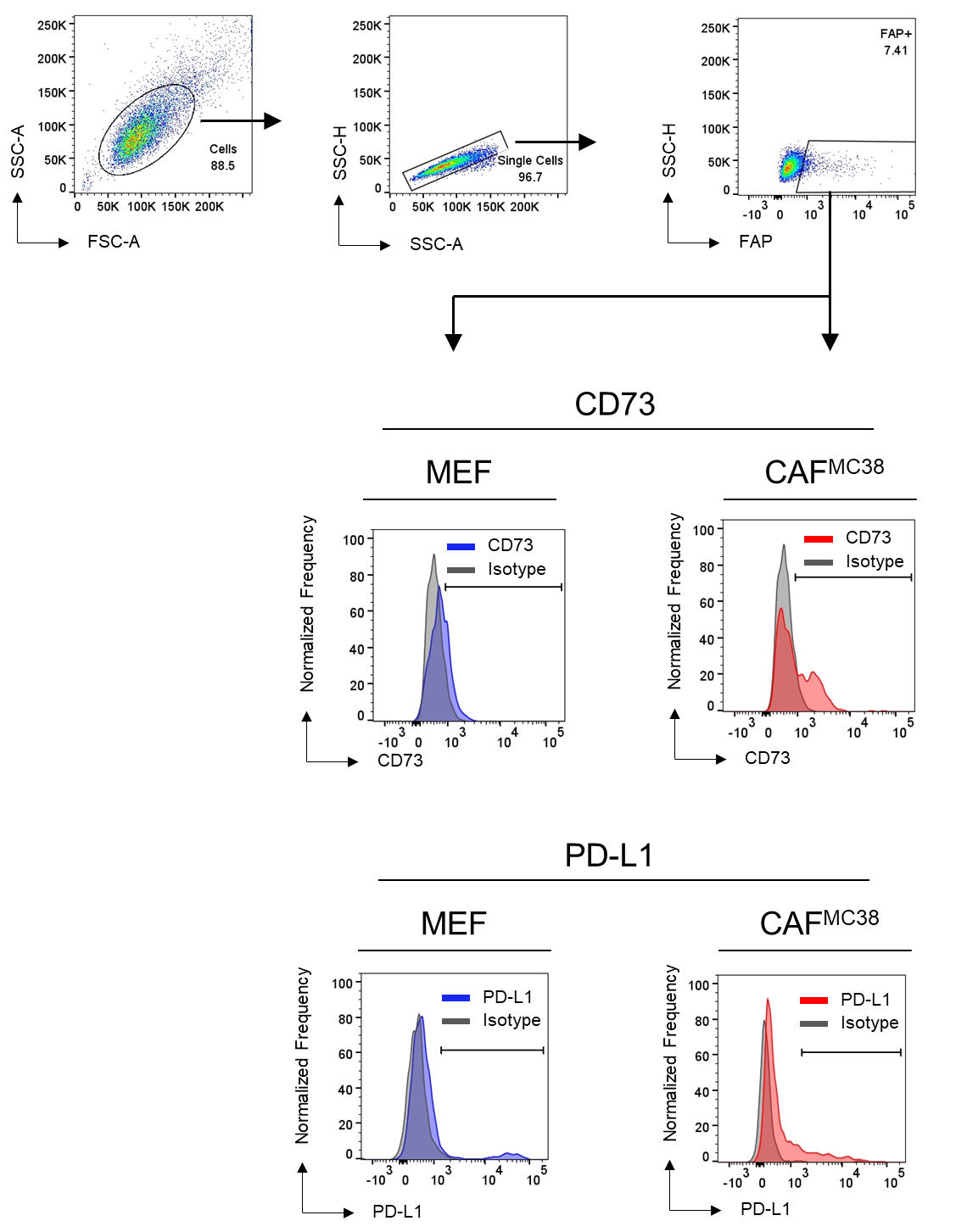


**Supplementary Figure S4. Gating strategy and expression of CD73 and PD-L1 for CAFs and normal fibroblasts by flow cytometry.**

Gating strategy and representative flow cytometry plots of MEF and CAF^MC38^ cells with isotype controls in vitro.

**Supplementary Figure S5**


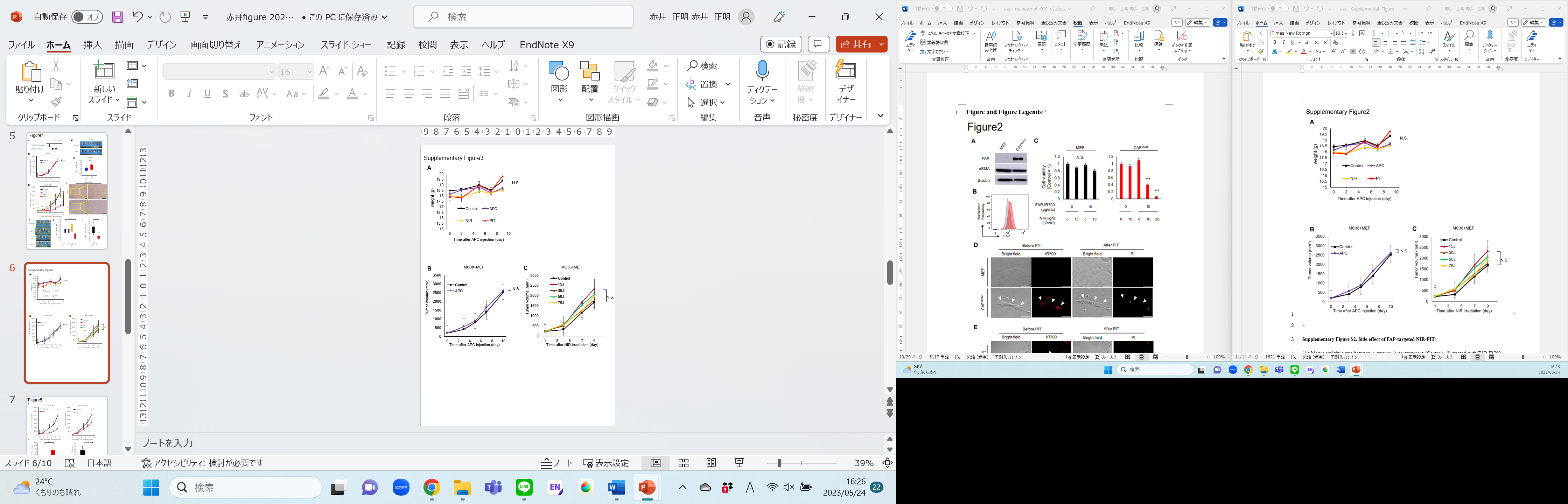


**Supplementary Figure S5. Side effect of FAP-targeted NIR-PIT**

(A) Mouse weight curve between 4 groups; ⅰ) no treatment (Control), ⅱ) treated with FAP-IR700 (APC), ⅲ) irradiated with NIR light (NIR), and ⅳ) treated with FAP-IR700 and irradiated with NIR light (PIT) on MC38+MEF tumors (n = 5) (N.S.; not significant; mean ± SEM; one-way ANOVA followed by Turkey’s at 9 d).

(B) Tumor growth curve between control and APC on MC38+MEF tumor (n = 4) (N.S.; not significant; mean ± SEM; one-way ANOVA followed by Turkey’s at 9 d).

(C) Tumor growth curve between control and NIR on MC38+MEF tumor (n = 4) (N.S.; not significant; mean ± SEM; one-way ANOVA followed by Turkey’s at 9 d).

**Supplementary Figure S6**


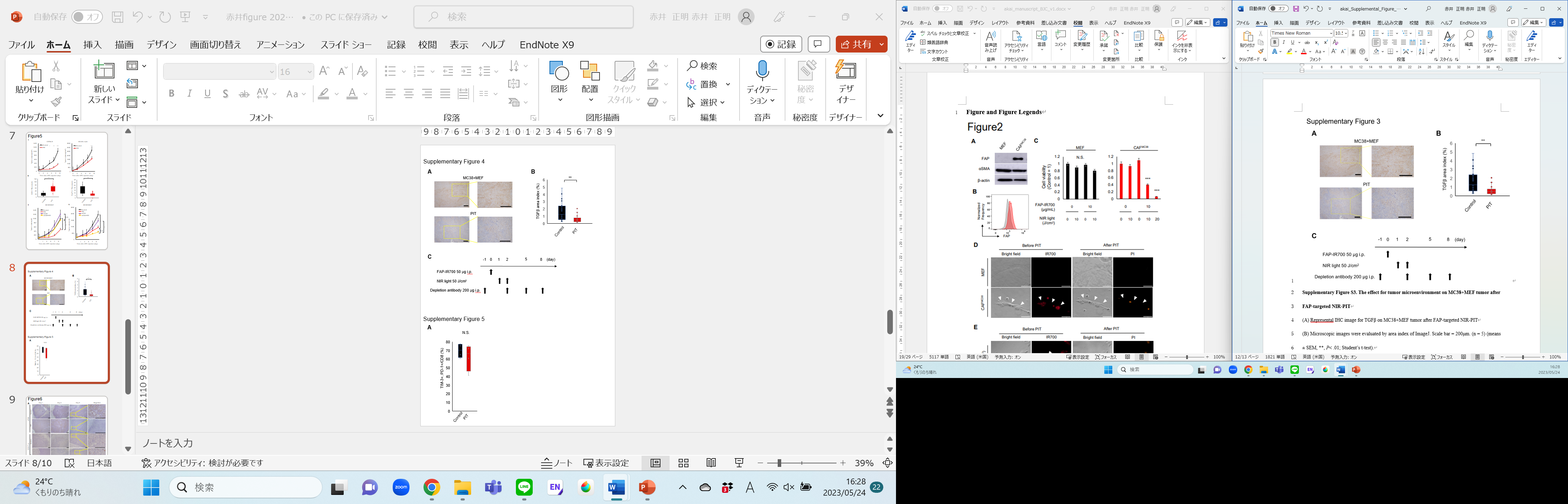


**Supplementary Figure S6. The effect for tumor microenvironment on MC38+MEF tumor after FAP-targeted NIR-PIT**

(A) Representative IHC image for TGF-β on MC38+MEF tumor after FAP-targeted NIR-PIT

(B) Microscopic images were evaluated by area index of ImageJ. Scale bar = 200 μm. (n = 5) (means ± SEM, **, *P*< .01; Student’s t-test).

(C) Therapy protocol with depletion antibody.

**Supplementary Figure S7**

**
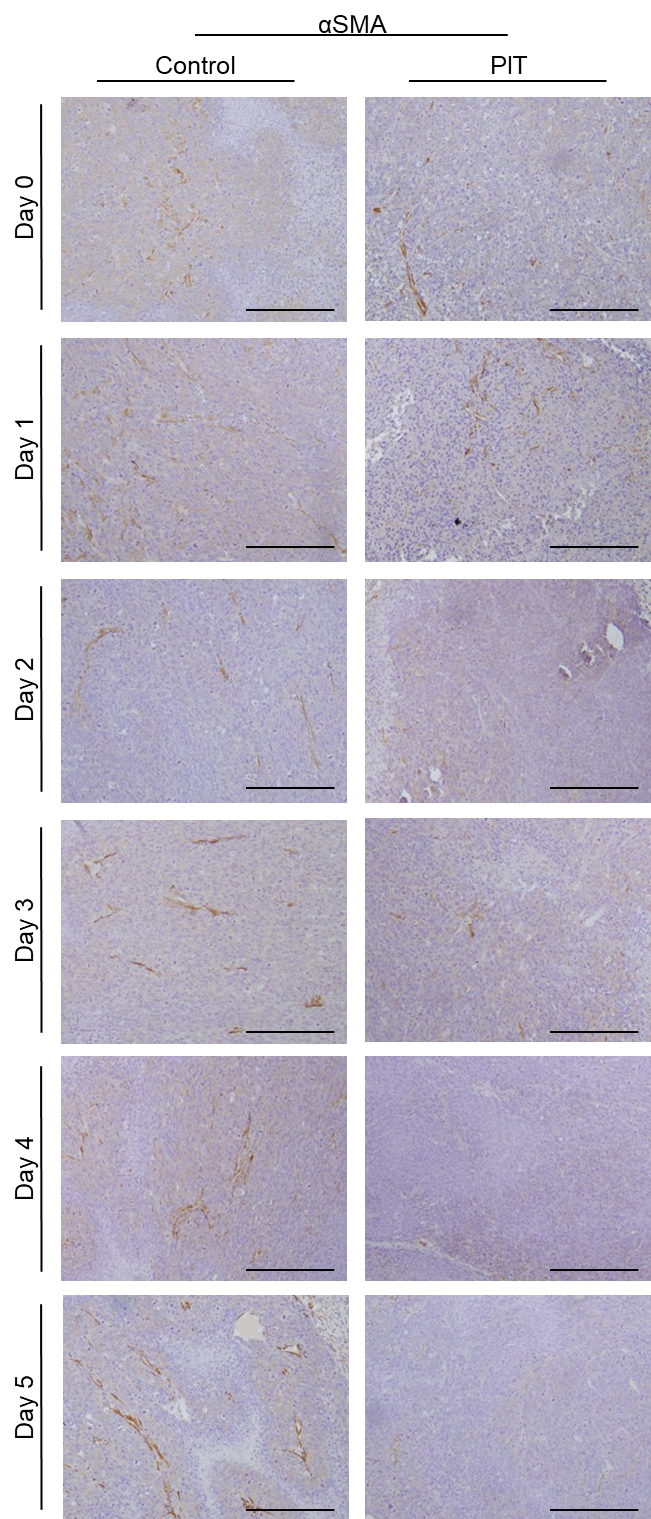
**

**Supplementary Figure S7. Representative immunohistochemical images of αSMA in the Control and PIT groups with several time course (day 0 to 5) in MC38+MEF allograft models.** Scale bar = 100 μm.

**Supplementary Figure S8**

**
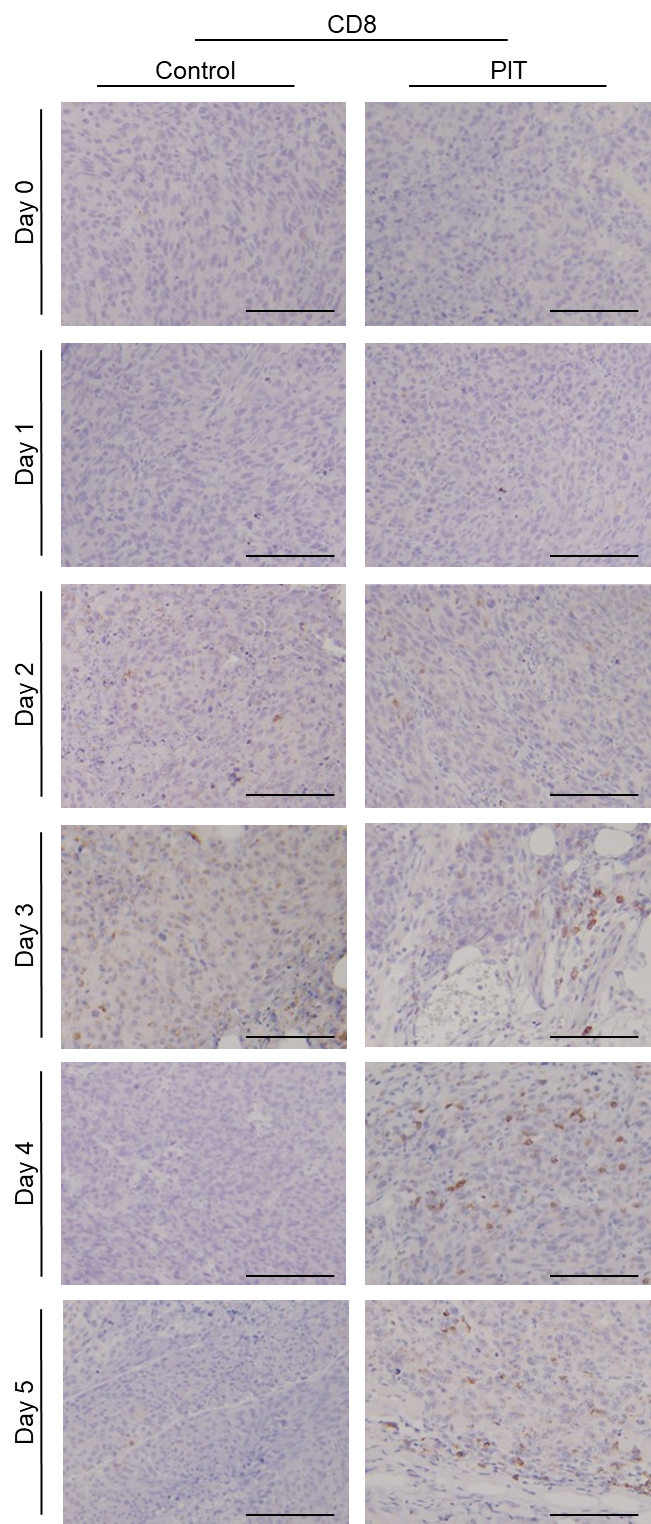
**

**Supplementary Figure S8. Representative immunohistochemical images of CD8^+^TILs in the Control and PIT groups with several time course (day 0 to 5) in MC38+MEF allograft models.** Scale bar = 50 μm.

**Supplementary Figure S9**

**
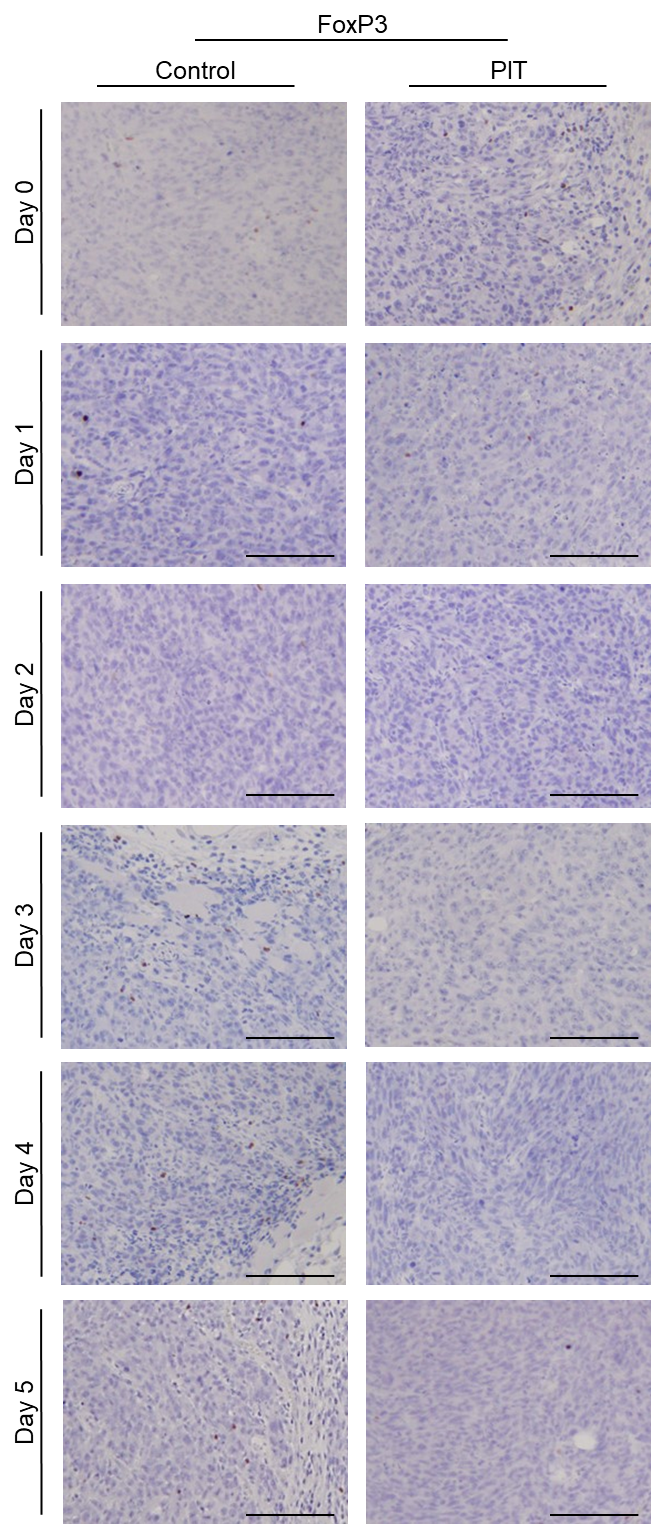
**

**Supplementary Figure S9. Representative immunohistochemical images of FoxP3^+^TILs in the Control and PIT groups with several time course (day 0 to 5) in MC38+MEF allograft models.** Scale bar = 50 μm.

**Supplementary Figure S10**


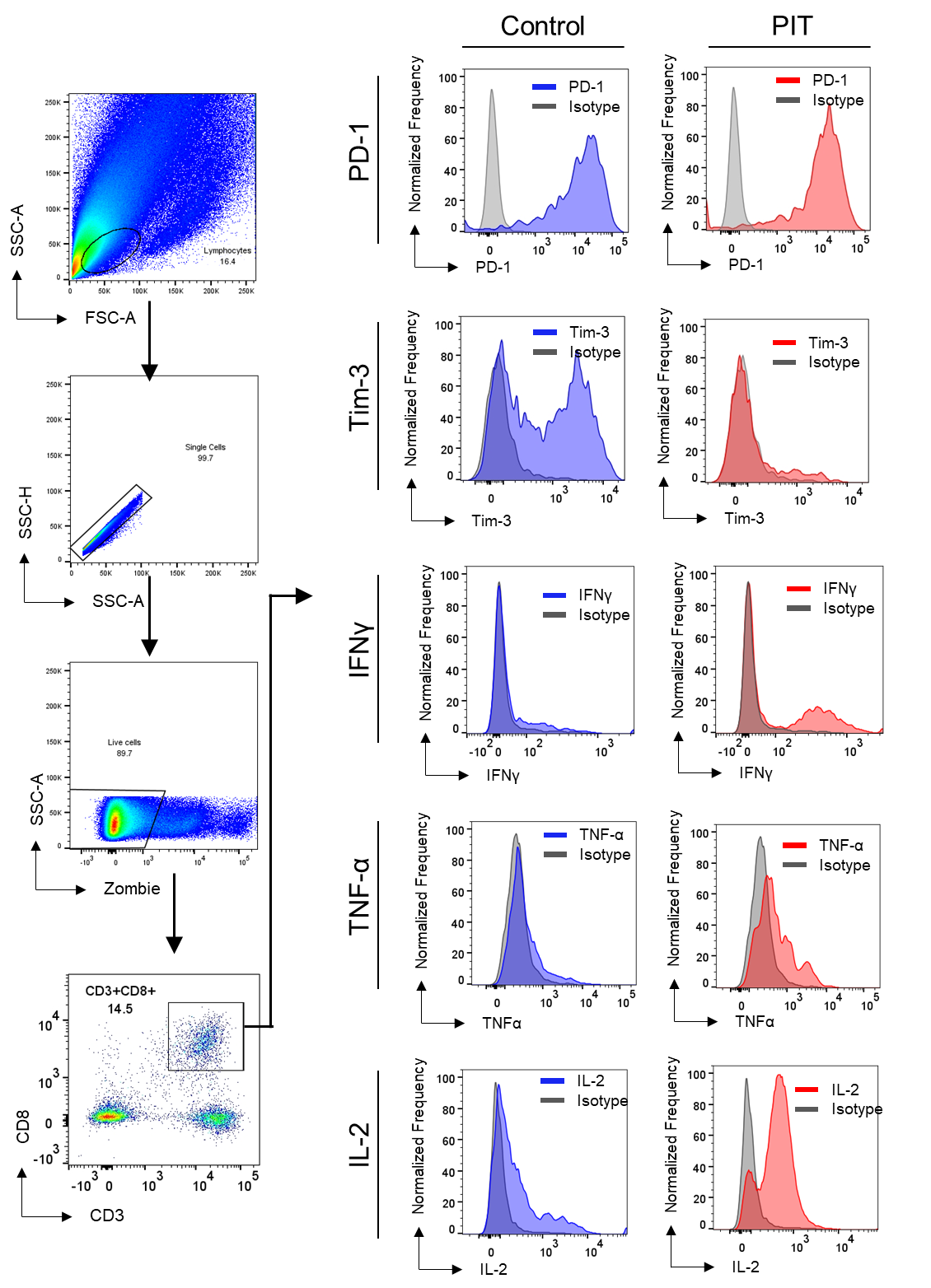


**Supplementary Figure S10. Gating strategy and representative flow cytometric histograms.**

Gating strategy and representative flow cytometric histgrams of tumor infiltrating CD8^+^ T cells with isotype controls in allograft models (control and PIT groups).

**Supplementary Figure S11**


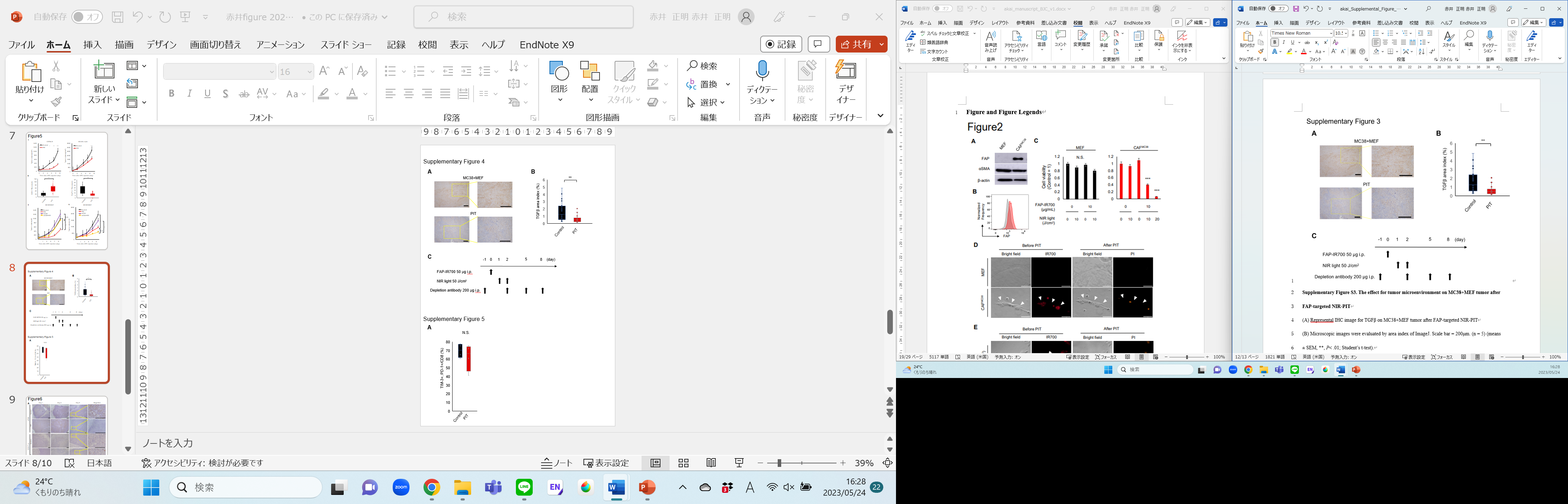


**Supplementary Figure S11. The effect for tumor infiltrating lymphocytes on MC38+MEF tumor after FAP-targeted NIR-PIT**

Flow cytometry analysis of TILs on MC38+MEF tumor after FAP-targeted NIR-PIT. The percentage of PD-1 (CD279)^+^ CD8^+^ TILs between control and FAP-targeted NIR-PIT (n = 4) (means ± SEM, N.S.; not significant; Student’s t-test).
